# RNF168 facilitates proliferation and invasion of esophageal carcinoma, possibly via stabilizing STAT1

**DOI:** 10.1111/jcmm.14063

**Published:** 2018-12-03

**Authors:** Na Yu, Min Xue, Weilong Wang, Dongxue Xia, Yajie Li, Xiaofeng Zhou, Dan Pang, Kui Lu, Jinghan Hou, Aijia Zhang, Ting Zhuang, Lidong Wang, Tingmin Chang, Xiumin Li

**Affiliations:** ^1^ Department of Gastroenterology the Third Affiliated Hospital of Xinxiang Medical University Xinxiang Henan P.R. China; ^2^ Center for Cancer Research Xinxiang Medical University Xinxiang Henan P.R. China; ^3^ Xinxiang Key Laboratory for Molecular Therapy of Cancer Xinxiang Medical University Xinxiang Henan P.R. China; ^4^ Institute of Lung and Molecular Therapy (ILMT) Xinxiang Medical University Xinxiang Henan Province P.R. China; ^5^ Laboratory of Molecular Oncology Henan Collaborative Innovation Center of Molecular Diagnosis and Laboratory Medicine, School of laboratory Medicine, Xinxiang Medical University Xinxiang Henan Province P.R. China; ^6^ Henan Key Laboratory for Esophageal Cancer Research The First Affiliated Hospital of Zhengzhou University Zhengzhou Henan Province P.R. China; ^7^ Department of Gastroenterology the First Affiliated Hospital of Xinxiang Medical University Weihui Henan P.R. China

**Keywords:** esophageal cancer, RNF168, stability, STAT1

## Abstract

Oesophageal cancer ranks as one of the most common malignancy in China and worldwide. Although genome‐wide association studies and molecular biology studies aim to elucidate the driver molecules in oesophageal cancer progression, the detailed mechanisms remain to be identified. Interestingly, RNF168 (RING finger protein 168) shows a high frequency of gene amplification in oesophageal cancer from TCGA database. Here, we report an important function for RNF168 protein in supporting oesophageal cancer growth and invasion by stabilizing STAT1 protein. RNF168 gene is amplified in oesophageal cancer samples, which tends to correlate with poor prognosis. Depletion RNF168 causes decreased cell proliferation and invasion in oesophageal cancer cells. Through unbiased RNA sequencing in RNF168 depleted oesophageal cancer cell, we identifies JAK‐STAT pathway is dramatically decreased. Depletion RNF168 reduced JAK‐STAT target genes, such as IRF1, IRF9 and IFITM1. Immuno‐precipitation reveals that RNF168 associates with STAT1 in the nucleus, stabilizing STAT1 protein and inhibiting its poly‐ubiquitination and degradation. Our study provides a novel mechanism that RNF168 promoting JAK‐STAT signalling in supporting oesophageal cancer progression. It could be a promising strategy to target RNF168 for oesophageal cancer treatment.

## 1 INTRODUCTION

Oesophageal cancer ranks no. 8 in malignancy incidence worldwide. There are around 500 000 diagnosed oesophageal cancer patients worldwide,^1^ while 60% of newly diagnosed cases are in China (477 900 cases).^2^ However, oesophageal squamous cell carcinoma is the major subtype in China, while oesophageal adenocarcinoma is the major subtype in western countries. Several environmental factors, such as smoking, hot drinks and alcohol, were found for oesophageal carcinoma.^3^ Besides, whole genomic sequencing revealed high rate of genetic events, including mutation and amplification, clustered in several important pathways such as proliferation, apoptosis and DNA repair control signalling.^4^ However, as a lack of genetic animal models in oesophageal cancer, the detailed carcinogenic mechanism and pathologic process are poorly understood.

The JAK‐STAT signalling (Janus kinase‐Signal transducer and activator of transcription proteins) is proved to involve in the process of immunity, development and human cancers.^5^ When JAK‐STAT signalling is activated, the ligands cause the dimerization of receptors, trans‐phosphorylation effect on JAK and subsequently promote STATs phosphorylation. The activated STATs form dimers, move from cytoplasm to the nuclear and promote the transcription of certain target genes.^6^ JAK‐STAT signalling was proved to involve in several human cancers, including oesophageal cancer. For example, the activation of JAK‐STAT via IL6 could promote oesophageal cancer cell survival in paracrine manner.^7^ EGFR signalling could also trans‐activate JAK‐STAT and promote oesophageal cancer cell migration.^8^ Besides, the ligands activation and cross‐talk with other signalling, little is known about the potential mechanisms and insights into novel components of JAK‐STAT signalling in dealing with oesophageal cancer progression.

There are about 700 of putative E3 ligases in humans, while the RING (Really Interesting New Gene) finger protein family attracted the research attention for the E3 ligase scientists due the non‐degradative ubiquitination function and modulation in chromatin structure.^9^ RNF168 (RING finger protein 168) was firstly characterized in 2009 as a novel ubiquitin interaction protein.^10^ Further studies demonstrated that RNF168 localizes in the nuclear and plays an essential role of ubiquitination in DNA damage response (DDR).^11^, ^12^, ^13^ In several DDR process, RNF168 locates at DNA repair sites and promotes mono‐ubiquitination of H2A/H2AX at K13‐15 to drive DNA repair complex formation.^11^ Previous studies have shown that RNF168 could participate in chemotherapy resistance in a group of malignancies.^14^ In our analysis, the public cancer database shows RNF168 has gene amplification in oesophageal cancer patients, which tends to correlate with poor overall survival. The current study reveals the function of RNF168 in promoting JAK‐STAT signalling in oesophageal cancer progression.

## 2 MATERIALS AND METHODS

### 2.1 Cell culture

NEC and EC109 oesophageal squamous cancer cells were acquired from previous study.^15^ NEC cell was cultured in DMEM (Invitrogen, Carlsbad, CA, USA) with 10% foetal bovine serum (FBS) and 1% penicillin/streptomycin (Invitrogen) at 37°C in a humidified atmosphere of 5% CO_2_ in air. EC109 cells were cultured in RPMI 1640 (Invitrogen) supplemented with 10% FBS (Gibco, Shanghai, China) and 1% penicillin/streptomycin. HEK293 cells were cultured in RPMI 1640 (Invitrogen) supplemented with 10% FBS (Gibco) and 1% penicillin/streptomycin.

### 2.2 siRNA and transfection

Cells were transfected with 50 nmol/L siRNA. RNF168 siRNAs sequences were shown here: RNF168 siRNA #1: 5‐CACAAAGCAUCCAACACCAdTdT‐3; siRNA #2: 5‐GAAGAUAUGCCGACACUUUdTdT‐3. Control siRNA sequences were shown: UUCUCCGAACGUGUCACGUTT. INTERFERin transfection reagent (Polyplus Transfection, 409‐10) was applied to the standard method. Lipofectamin 2000 (1662298; Invitrogen) was used for plasmid tranfection.

### 2.3 Plasmids

Flag‐STAT1 and MYC‐RNF168 plasmids were acquired from the Origene Company (Beijing, China) and were described in the previous study.^16^

### 2.4 RNA extraction and qPCR analysis

RNA extraction process was described in the previous study.^17^ 36B4 was utilized for internal control. Primer sequences for qPCR are provided in Table 1.

**Table 1:** Primer sequences for Q‐PCR

Primer for Q‐PCR	
RNF168 Forward primer	ggc gag ttt atg ctg tcc ct
RNF168 Reverse primer	gcc gcc acc ttg ctt att tc
36B4 Forward primer	ggc gac ctg gaa gtc caa ct
36B4 Reverse primer	cca tca gca cca cag cct tc
STAT1 Forward primer	gcc aag caa gaa agt gtc ct
STAT1 Reverse primer	ctg aat att tcc ctc ctg gg
IRF1 Forward primer	caa ctt cca ggt gtc acc ca
IRF1 Reverse primer	cga ctg ctc caa gag ctt ca
IFITM1 Forward primer	ccg tga agt cta ggg aca gg
IFITM1 Reverse primer	ggt aga ctg tca cag agc cg
IRF9 Forward primer	ggg gag cag tcc att cag ac
IRF9 Reverse primer	gca gtg agt agt ctg gct ctg

### 2.5 Quantification of cell viability

RNF168 siRNA were transfected into NEC/EC109 cells, while siControl were used as negative control. After 24 hours, the cells were seeded with equal amount into 96‐well plate. The cell viability was determined by WST‐1 reagent, which was described in previous study.^18^

### 2.6 Western blotting

RIPA lysis buffer was used to extract protein from cell lysis. Anti‐Tubulin mouse (T5168) was acquired from Sigma (Wuxi City, China). Anti‐actin (8H10D10) was acquired from Cell Signaling Technology (Beijing, China). Anti‐RNF168 (SC‐101125) was from Santa Cruz Biotechnology (Shanghai, China). Anti‐STAT1 (9172) was acquired from Cell Signaling Technology. anti‐Lamin B1 (66095‐1) was acquired from Wuhan Sanyin company (Wuhan, China).

### 2.7 Co‐immunoprecipitation

Co‐immunoprecipitation experiment was described in previous study.^19^ Mouse Ig G was used to pre‐clear the unspecific protein binding. Cell lysates were pre‐cleared for 2 hours and subsequently incubated overnight with RNF168 mouse antibody (SC‐101125), while mouse IgG was used as negative control. The lysis were washed and analysed by immuno‐blot with STAT1 antibody (9172).

### 2.8 Protein stability assays

The STAT1 protein stability was determined between siControl and siRNF168 group in NEC cells. After 24 hours of siRNA transfection, cells were treated with cycloheximide (100 μmol/L) at indicated time points. Samples were analysed by immune‐blot for STAT1 protein level.

### 2.9 Wound healing assay

NEC cells were used for wound healing assay. Cells were transfected with RNF168 siRNA and siControl. After 24 hours, cells were treated with trypsin and seeded into six‐well plate with 1% FBS with 100% confluence. The yellow tip was applied for making a straight line. The distance was determined at indicated time points. Percentage wound recovery was calculated as: [1−(Width of the wound at a given time/width of the wound at *t* = 0)] × 100%.

### 2.10 Trans‐well invasion assay

The transwell invasion assay was performed with two‐chamber plates (#3422; Corning, Shanghai, China). For the transwell assay, 1 × 10^5^ NEC or EC109 control cells and RNF168 knocking‐down cells were seeded into the top chamber. To stimulate the invasion, medium with 10% FBS was added to the bottom wells. Twenty‐four hours post‐incubation, cells in the upper chamber were washed out and the cells that had migrated into the membrane were fixed and stained with crystal violet staining solution. Cellular quantification was analysed in three random fields with 100‐fold magnification under microscope. The quantification was based on cell number counting in each vision.

### 2.11 Analysis of protein ubiquitination

One million HEK293 cells were seeded into 10 cm plate. After 24 hours, cells were transfected with 4 μg pCMV‐myc‐STAT1 together with 4 μg pCDNA3‐Flag‐RNF168. After 48 hours, cells were treated with 10 μmol/L MG132 or ethanol for 8 hours. The ubiquitinated STAT1 were detected by Western blot analysis.

### 2.12 RNA sequence analysis

The whole genomic expression profiling analysis was based on RNA sequencing platform from BGI (Beijing Genomic Institute). The RNA sequence data are deposited in the Gene Expression Omnibus (GEO) database (assessing number: GSE118532). Differentially expressed genes was extracted for analysis (*P* value < 0.01 together with fold change >2) by Ingenuity Pathway Analysis.

### 2.13 Statistics

Student's *t* test and Pearson correlation coefficient were used for comparisons. For multiple group comparison, ANOVA (analysis of variance) was used for comparisons. Tukey's test was used as the post‐hoc test after ANOVA text. *P* < 0.05 was considered to be significant.

## 3 RESULTS

### 3.1 RNF168 has high frequency of gene amplification in oesophageal cancer and tends to correlate with poor prognosis in oesophageal cancer patients

**Figure 1:**
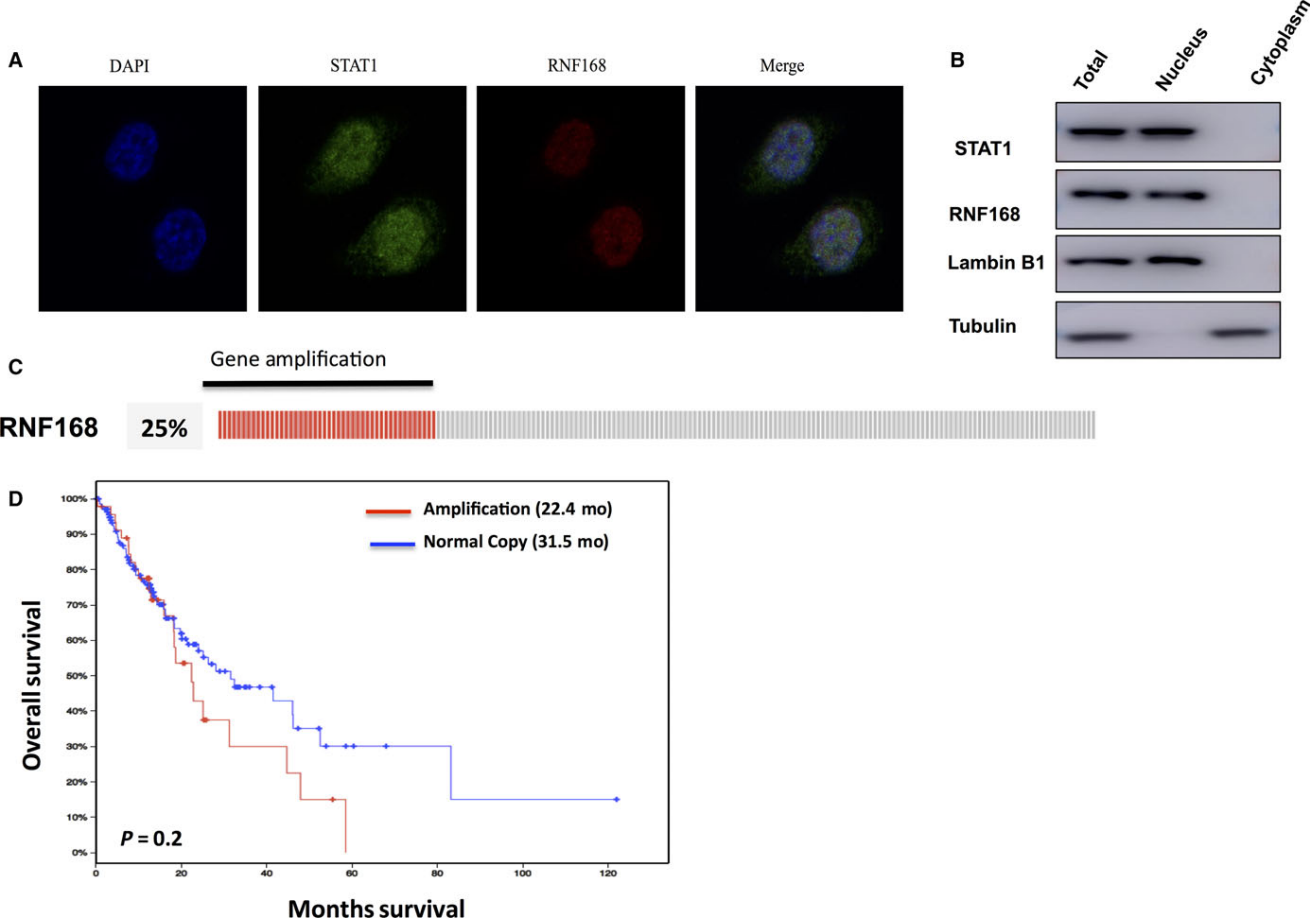
RNF168 has high frequency of gene amplification in oesophageal cancer and tends to correlate with poor prognosis in oesophageal cancer patients. (A) NEC cells cultured on sterile glass cover slips were fixed with 4% formaldehyde for 10 min. Then the cells were incubated in permeabilization buffer (0.3% Triton X100, in phosphate buffered saline [PBS]) for 10 min. Ten percent donkey serum was added to suppress nonspecific antibody binding at room temperature for 30 min and primary antibodies were incubated overnight at 4°. Fluorochrome‐conjugated secondary antibodies were added after wash in a dark chamber at room temperature. The slides were washed with PBS and mounted using mounting solution containing DAPI. Finally, slides were visualized with a NIKON80i fluorescent microscope. The antibodies were used as follows: anti‐RNF168 (SC‐101125, santa cruz); anti‐STAT1 (9172, Cell Signalling Technology). (B) RNF168 is mainly localized in the nuclear. The subcellular protein fractionation kit (Thermo Scientific, 78840) was used for cytoplasm and nuclear separation. Tubulin and Lambin B1 were used for cytoplasm and nuclear control. (C, D) RNF168 has gene amplification in 25% of esophageal cancer patients. The TCGA database was used to extract the gene expression data. RNF168 gene amplification tends to correlate with poor overall survival in esophageal cancer patients

Firstly, we identify the localization of RNF168 in oesophageal cancer cells. The immune‐staining results indicate RNF168 is located in the nucleus in NEC cells (Figure 1A). The cytoplasmic and nucleus separation assay shows that RNF168 is mostly localized in the nuclear in NEC cells (Figure 1B). The TCGA data (The Cancer Genome Atlas) shows that RNF168 gene is amplified in 25% of oesophageal cancer samples (Figure 1C). Through the survival analysis from TCGA database, we observe that RNF168 gene amplification is correlated with poor overall survival in oesophageal cancer patients (http://www.cbioportal.org/study?id=esca_tcga#summary), which may indicate the involvement of RNF168 in oesophageal cancer progression (Figure 1D).

### 3.2 RNF168 knocking down inhibits cell proliferation and invasion in oesophageal cancer cells

**Figure 2:**
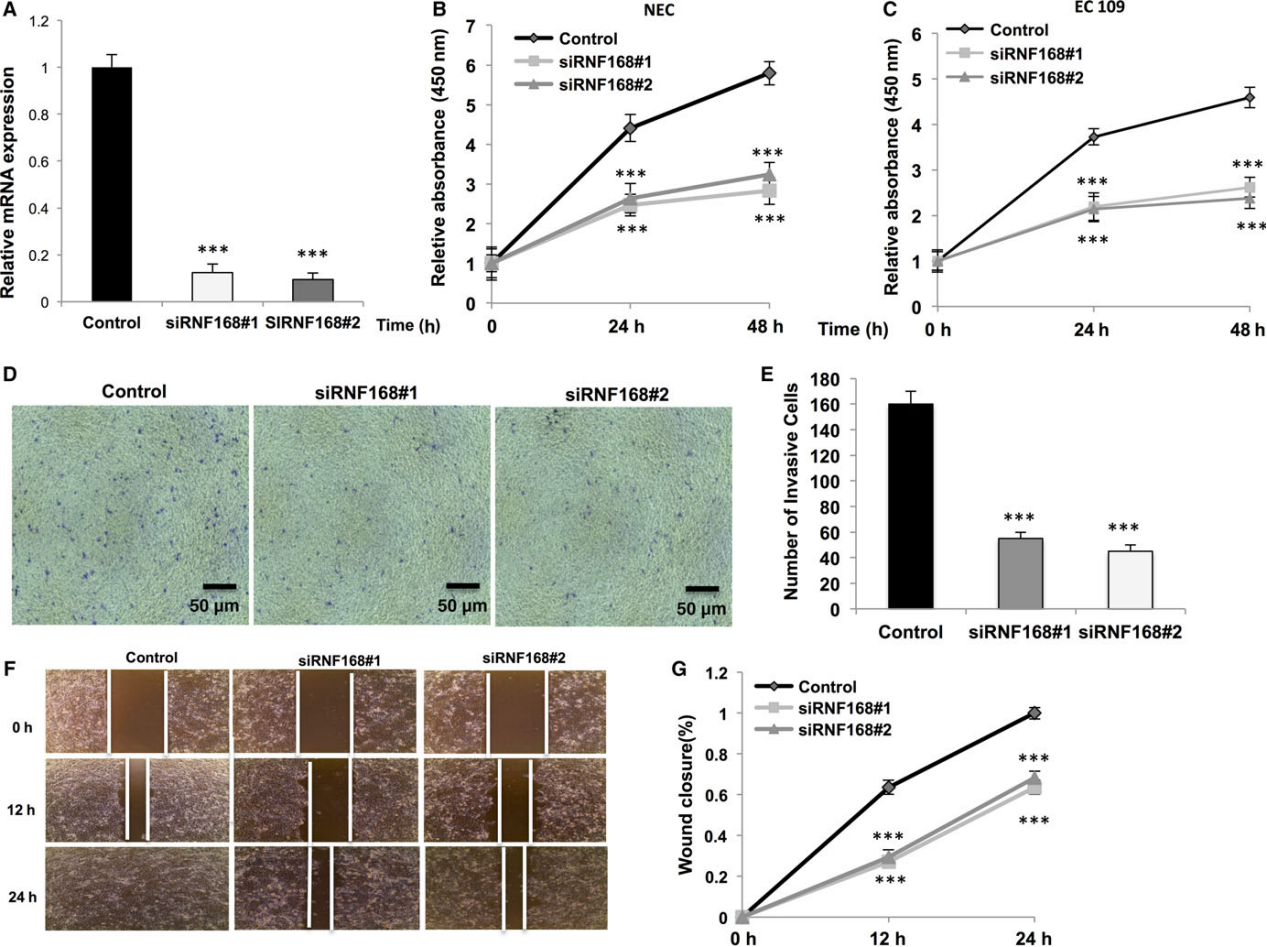
RNF168 depletion inhibits cell proliferation and invasion in oesophageal cancer cells. (A) RNF168 depletion effect by two different siRNA oligos. NEC cell were transfected with siRNF168 or siControl. After 48 h, RNF168 mRNA levels are determined by real‐time PCR with 36B4 as internal control. (B, C) The WST‐1 assay was used to determine the cellular metabolic activity at indicated time points after transfection. NEC and EC109 cells were transfected with siRNF168 and siControl. After 24 h, cells were seeded into 96‐well plates. These experiments were done in triplicates. All values are mean ± SD (n = 3, *P <* *0.05; **P < 0.01, ***P < 0.001). (D, E) Transwell invasion assay of NEC cells transfected with indicated RNF168 siRNA. The cell number is counted and data are presented as ±SD. **, *P* < 0.01, ***, *P* < 0.001 (Student's *t* test). (F, G) Wound healing assay of NEC transfected with the indicated siRNA. Quantification of wound closure at the indicated time points. Data are presented as ±SD. **, *P* < 0.01, ***, *P* < 0.001 (Student's *t* test)

In order to investigate RNF168 function in oesophageal cancer, we deplete RNF168 expression by two individual siRNAs (Figure 2A). RNF168 depletion via two independent siRNAs significantly inhibits cell growth in both NEC and EC109 cells by WST‐1 assay (Figure 2B,C). Besides, trans‐well assays indicate RN168 depletion significantly inhibits cell migration capacity in both NEC and EC 109 cells (Figure 2D,E, Fig. S1A). In parallel, the wound‐healing assay shows RNF168 depletion significantly decreases oesophageal cancer cell migration capability (Figure 2F,G). All these experiments indicate that RNF168 is essential for oesophageal cancer cell progression.

### 3.3 RNF168 depletion decreases STAT1 protein level and JAK‐STAT target genes in oesophageal cancer cells

**Figure 3:**
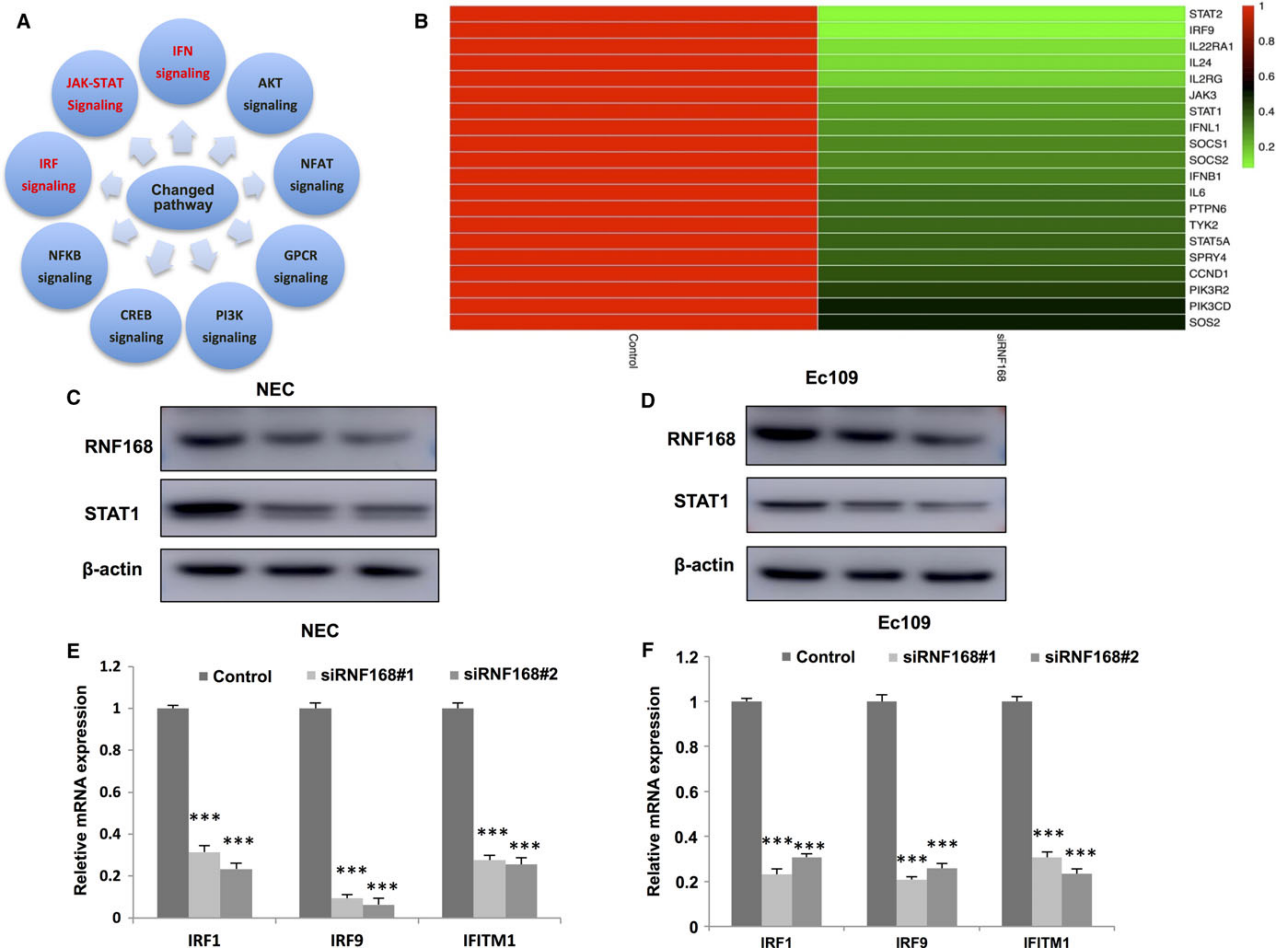
RNF168 depletion decreases STAT1 protein level and JAK‐STAT target genes in esophageal cancer cells. (A) Top 10 signalling pathways significantly decreased by RNF168 depletion in NEC cells. The pathway‐enrichment analysis was used by the threshold *P* < 0.001 and fold change >2 to derive regulated genes. RNF168 was depleted by siRNA (mix of siRNF168 #1 and siRNF168 #2) or treated with siControl. After 48 h, the whole mRNA was extracted for RNA sequence analysis. The siControl and siRNF168 were done in triplicates. B: The heat‐map graph shows the JAK‐STAT target genes, which is significantly decreased by RNF168 depletion in NEC cells. The significantly regulated genes were overlapped with publish JAK‐STAT target gene data. (C, D) RNF168 depletion effect on STAT1 protein level by two different siRNA oligos. NEC and EC109 cells were transfected with siRNF168 or siControl. After 48 h, RNF168 and STAT1 protein levels were determined by Western blot analysis. Actin was used as internal control. (E, F) RNF168 depletion decreases STAT1 target genes using two different siRNA oligos. NEC and EC109 cells were transfected with siRNF168 or siControl. After 48 h, cells, total RNA was prepared and the expression of the endogenous STAT1 target genes, IRF1, IRF9 and IFITM1 were determined by qPCR. Shown are the results from three experiments. *P < 0.05; **P < 0.01; ***P < 0.001 for target gene expression comparison

To investigate the function of RNF168 in oesophageal cancer cells in an unbiased approach, we carry out the whole genomic profiling based RNA sequence by comparison between control and RNF168 depletion in NEC cells. By comparison with control cells, RNF168 depletion is associated with changes in several pathways, including IRF signalling (Interferon regulation factor), JAK‐STAT signalling and IFN signalling (Interferon), which share the STAT proteins as the common pathway effectors (Figure 3A). By comparing with published JAK‐STAT target genes with our different expressed genes by RNF168 depletion, 20 JAK‐STAT target genes are significantly down‐regulated, suggesting the potential regulation of RNF168 in JAK‐STAT signalling (Figure 3B). Since the STATs proteins are the effectors in target gene regulation. We deplete RNF168 via two different siRNAs to observe STATs protein level. We observed that STAT1 protein level and mRNA level is decreased in both NEC and EC109 cells (Figure 3C,D). Beside, RNF168 depletion also dramatically decreases JAK‐STAT target gene expression, including IRF1, IRF9 and IFITM1 in NEC and EC109 cells (Figure 3E,F, Fig. S1B,C).

### 3.4 RNF168 associates with STAT1 in the nuclear and increases STAT1 protein stability

**Figure 4:**
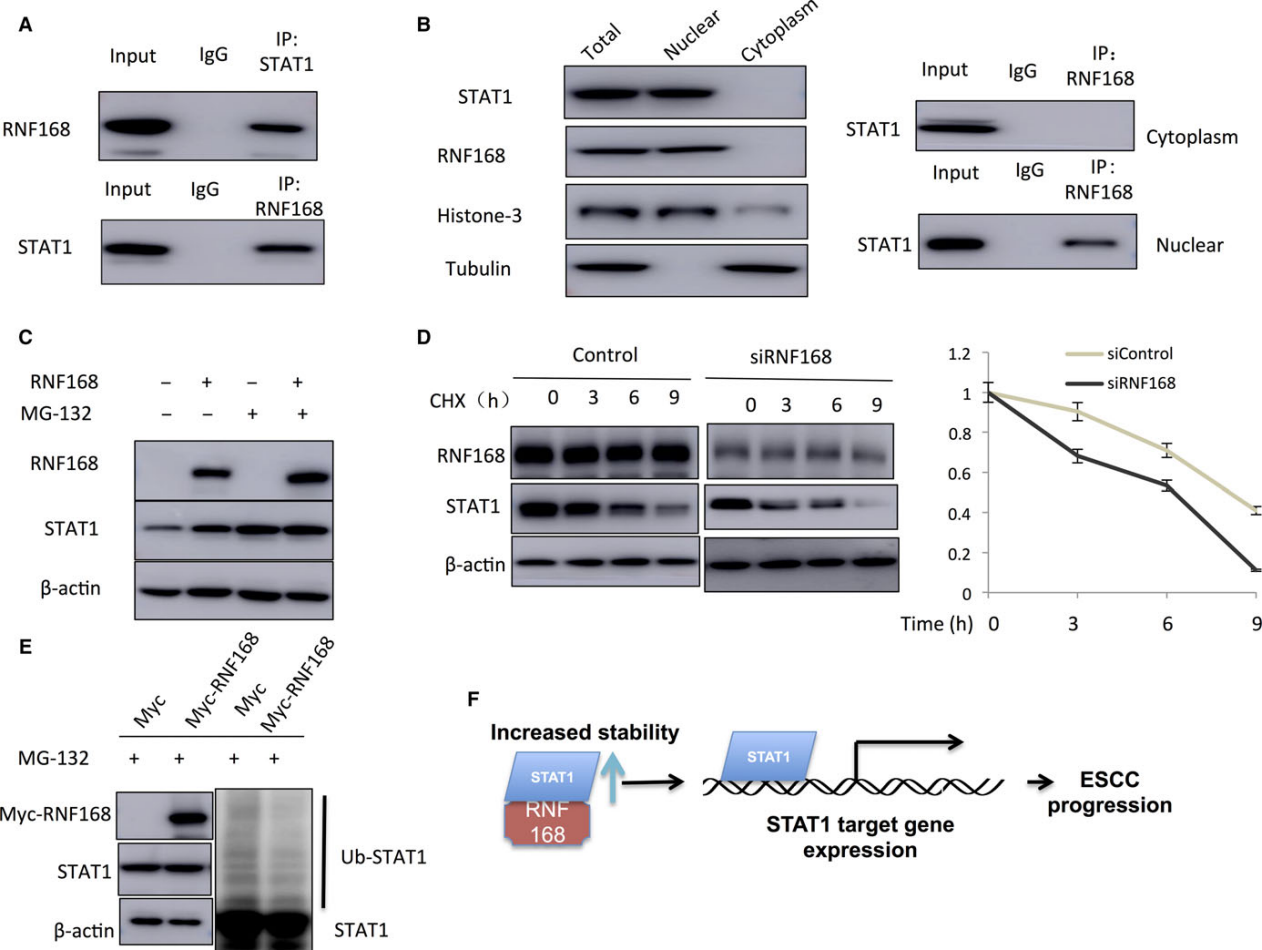
RNF168 associates with STAT1 in the nuclear and increases STAT1 protein stability. (A) Co‐IP assay reveals association between endogenous RNF168 and STAT1 in NEC cells. NEC cells were harvested with NP‐40 lysis buffer. CO‐IP was performed using antibody as indicated. (B) RNF168 is mainly localized in the nuclear and associates with STAT1 in the nuclear. The subcellular protein fractionation kit (Thermo scientific, 78840) was used for cytoplasm and nuclear separation. Tubulin and Histone‐3 were used for cytoplasm and nuclear control. Based on the separation, IP was done by RNF168 antibody in both the cytosol and nuclear lysis. STAT1 antibody was used to detect the interaction in both the cytosol and nuclear. (C) In the presence of the proteasome inhibitor MG132, the stabilization effect of RNF168 on STAT1 did not further increase STAT1 protein levels. HEK293 cells were transfected with 2 μg STAT1 plasmid and 0.5 μg Myc‐tag or Myc‐RNF168 plasmids. After 24 h, cells were treated with 10 μmol/L MG132/vehicle for 6 h. Cell lysates were prepared for Western blot analysis. The results are representative for three independent experiments. (D) RNF168 increases STAT1 half‐life in NEC cells. NEC cells were transfected with 50 nmol/L siControl or siRNF168 siRNA. After 24 h, cells were treated with 100 μmol/L cycloheximide/vehicle for indicated times. Cell lysates were prepared for Western blot analysis. The results are representative for three independent experiments. The STAT1 relative density was measured by Image J software. (E) RNF168 prohibits STAT1 poly‐ubiquitination. HEK293 cells were transfected with 2 μg STAT1 plasmid and 0.5 μg Myc‐tag or Myc‐RNF168 plasmids. After 24 h, cells were treated with 10 μmol/L MG132 or vehicle for 6 h. Cells were directly harvested and Western blot analysis using STAT1 antibody was used to detect ubiquitinated STAT1 forms. The predicted molecular weight of polyubiquitinated STAT1 is indicated. (F) The hypothetical model for the regulatory mechanism in RNF168 on JAK‐STAT1 signalling in esophageal cancer cells: RNF168 associates with STAT1 in the nuclear and subsequently stabilizes STAT1 by inhibiting its polyubiqutination process

Since RNF168 is a putative E3 ligase, we infer that RNF168 might regulate STAT1 through regulating protein stability. Immuno‐precipitation shows that RNF168 associates with STAT1 in oesophageal cancer cells (Figure 4A). Nuclear‐cytoplasm separation based Immuno‐precipitation shows that RNF168 associates with STAT1 in the nucleus (Figure 4B). With the treatment of proteasome inhibitor MG132, the stabilized effect on STAT1 by RNF168 could be rescued by MG132, which indicates RNF168 modulates STAT1 stability (Figure 4C). Up on inhibition of protein synthesis by cycloheximide, the presence of RNF168 significantly increases the half‐life of STAT1 protein in NEC cells (Figure 4D). With the treatment of proteasome inhibitor MG132, we observe that the expected poly‐ubiquitin chains of STAT1 in the presence of RNF168 is significantly decreased (Figure 4E). These experiments show that RNF168 modulates STAT1 via increasing protein stability.

## 4 DISCUSSION

In the study, we show that the E3 ubiquitin ligase RNF168 promotes oesophageal cancer cell proliferation and invasion, possibly via activating JAK‐STAT signalling. Besides, we also observe that RNF168 gene is amplified in 25% of oesophageal cancer patients and tends to relate with shorter overall survival time. The unbiased RNA sequencing assay shows that RNF168 is required for JAK‐STAT signalling activity, while the mechanistic study shows that RNF168 associates with STAT1 in the nuclear, stabilizes STAT1 protein and inhibits its poly‐ubiquitination. This study provides a novel mechanism that RNF168 modulates JAK‐STAT signalling via regulating STAT1 protein stability in oesophageal cancer. On the basis of these knowledge, we propose that selective modulation of RNF168‐STAT1 interaction could be a promising strategy to inhibit oesophageal cancer proliferation and invasion (Figure 4F).

The JAK‐STAT1 signalling is composed of three core parts: Janus kinases (JAKs), STATs and receptors, while the STATs proteins are the effectors in nuclear shuttling and gene regulation. As the first identified STATs proteins, STAT1 involves in several important biological functions in normal cells, such as cell differentiation, immune system regulation.^20^ However, the function of STAT1 in cancers is controversial. STAT1 could promote the several apoptotic molecules, such as FAS/FASL to induce cell death in colon cancer and blood malignancies.^21^ Besides, the aberrant activation or expression of STAT1 has been detected some type of human cancers, including breast cancer, head and neck cancer and lymphoma.^22^, ^23^ When comes to oesophageal cancer, STAT1 was shown to promote invasion and migration of tumour cells, while depletion of tumour suppressor p19 led to increased tumour formation in nude mice.^24^ Based on the oncogenic role of STAT1 in oesophageal cancer, targeting STAT1 via various strategies could be promising in oesophageal cancer therapy.

Quite a few RING protein family members have been shown to modulate nuclear receptors and transcriptional factors via transcriptional or post‐translational regulation.^25^, ^26^, ^27^, ^28^, ^29^ For example, RNF8 and RNF31 were shown to promote oestrogen receptor alpha protein stability via facilitating ER alpha mono‐ubiquitination.^27^, ^29^, ^30^ RNF168 as a family of RING finger proteins, was firstly discovered as an ubiquitin‐binding domain protein.^10^ Further studies demonstrated that RNF168 plays an essential role of ubiquitination modification in DDR.^11^, ^12^, ^13^ In several DNA damage process, RNF168 is recruited to DNA damage foci and promotes mono‐ubiquitination of H2A/H2AX at K13‐15 to facilitate the formation of DNA repair complex.^11^ Previous studies showed that RNF168 could participate in chemotherapy resistance in various type of cancers.^14^ Interestingly, RNF168 is highly amplified in oesophageal cancer patients, which lead our interests to dig into the mechanisms. Depletion of RNF168 significantly hampers the proliferation and invasion capacity in oesophageal cancer cells. We further examine the role of RNF168 in STAT1 and its function. RNF168 is shown to associate with STAT1 protein and prolong its stability. In all, our study reveals an important cancer regulation mechanism between RNF168 and JAK‐STAT signalling, which indicates modulation of RNF168 protein could be an approach in inhibiting oesophageal cancer progression.

## ACKNOWLEDGEMENT

We thank all the members of Institute of Lung and Molecular Therapy (ILMT) for sharing valuable material and research support.

## AVAILABILITY OF DATA AND MATERIALS

Additional data and materials may be requested from the corresponding author on reasonable request.

## AUTHORS’ CONTRIBUTIONS

TMC, and XML contribute to the manuscript writing. NY, MX, WLW, DXX, YJL, XFZ, DP, KL, JHH and AJZ contribute to the molecular and cellular biology experiments. MX contributes to the clinical data analysis and RNA sequence data analysis. XML and LDW contribute to the funding support for this study.

## COMPETING INTEREST

The authors declare that they have no competing interests.

## ETHICS APPROVAL AND CONSENT TO PARTICIPATE

Not applicable.
