# Supplementary material for: RNF168 facilitates proliferation and invasion of esophageal carcinoma, possibly via stabilizing STAT1
Source: J Cell Mol Med. 2018 Dec 3;23(2):1553–61. doi: 10.1111/jcmm.14063 (PMC6349343; doi:10.1111/jcmm.14063)
Supplement: Supplementary file 1 [file JCMM-23-1553-s001.pptx]

## Slide 1
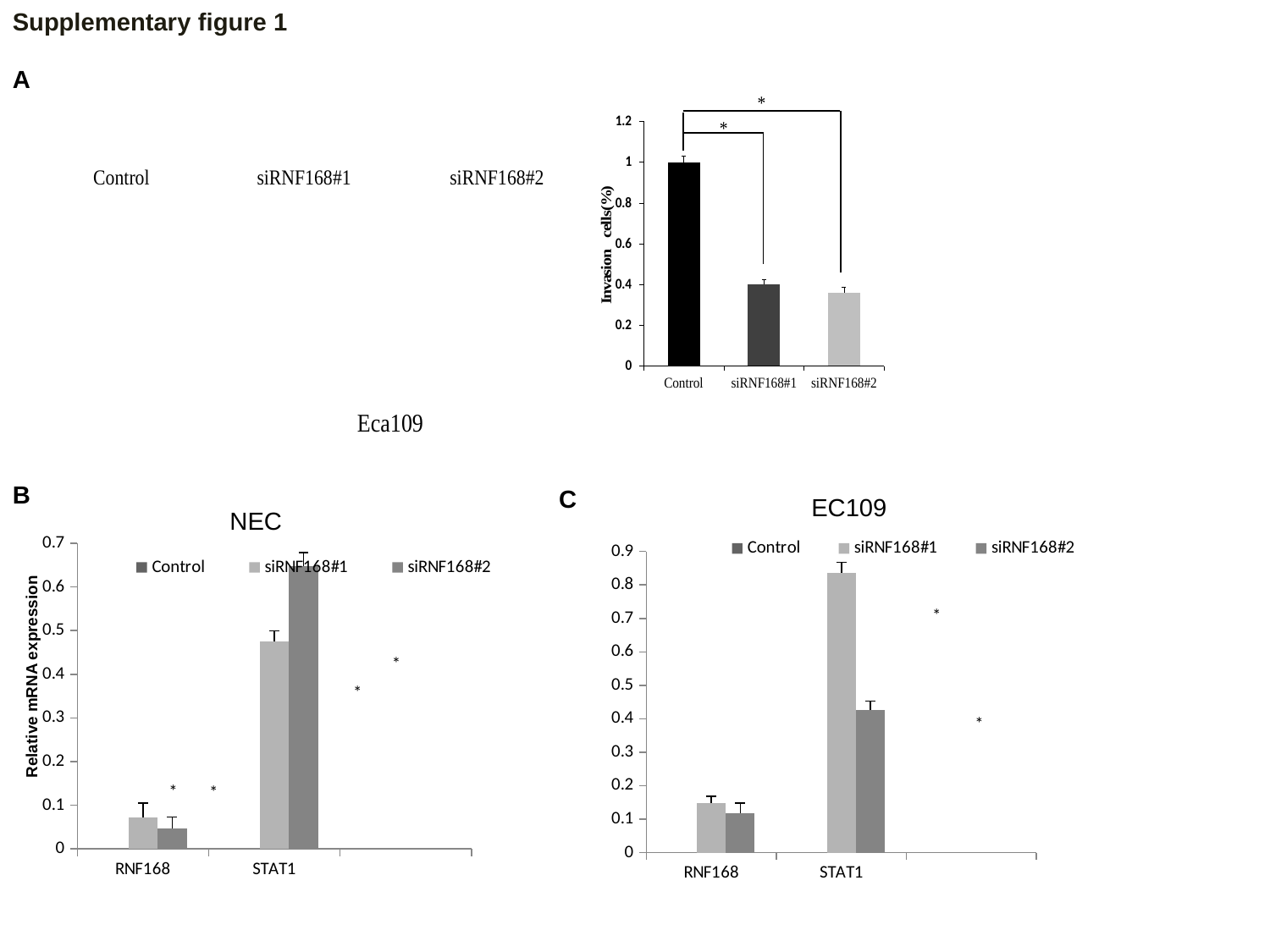

Supplementary figure 1
A
B
C
EC109
NEC
### Chart
| Category | Control | siRNF168#1 | siRNF168#2 |
|---|---|---|---|
| RNF168 | 1.0 | 0.146793182670011 | 0.117173633824336 |
| STAT1 | 1.0 | 0.836625232445289 | 0.42518277872761 |
### Chart
| Category | Control | siRNF168#1 | siRNF168#2 |
|---|---|---|---|
| RNF168 | 1.0 | 0.0724954292602094 | 0.046308128177248 |
| STAT1 | 1.0 | 0.47594904326493 | 0.647155417694915 |*
*
Relative mRNA expression
*
*
*
*
